# Deep Introspection Regarding Cumulative Prognostic Factors in Liposarcoma and Atypical Lipomatous Tumor

**DOI:** 10.3390/medicina61081431

**Published:** 2025-08-08

**Authors:** Ana-Maria Ciongariu, Șerban-Ștefan Radu, Adrian-Vasile Dumitru, Cătălin Cîrstoiu, Valentin Enache, Andrei Marin, Cosmin Creangă, Mariana Costache

**Affiliations:** 1 Department of Pathology, Faculty of Medicine, “Carol Davila” University of Medicine and Pharmacy, 030167 Bucharest, Romania; ana-maria.ciongariu@drd.umfcd.ro (A.-M.C.);; 2 Faculty of Medicine, “Carol Davila” University of Medicine and Pharmacy, 030167 Bucharest, Romania; raduserbanstefan@gmail.com; 3 Department of Orthopedy and Traumatology, “Carol Davila” University of Medicine and Pharmacy, 030167 Bucharest, Romania; cirstoiu_catalin@yahoo.com; 4 Department of Pathology, Clinical Emergency Hospital, 030167 Bucharest, Romania; valienache00@gmail.com (V.E.); coscrean@gmail.com (C.C.); 5 Department of Plastic Surgery, Faculty of Medicine, “Carol Davila” University of Medicine and Pharmacy, 030167 Bucharest, Romania; andrei.marin@umfcd.ro

**Keywords:** liposarcoma, grade, Ki67, prognosis, survival

## Abstract

*Background and Objectives*: Prognostic evaluation for patients with liposarcoma and atypical lipomatous tumor is a complex process, considering the marked heterogeneity of this group of mesenchymal neoplasms. At the moment, guidelines recommend determining the tumor’s histological grade by documenting proliferative activity and the presence of tumor necrosis. Proliferative intratumoral activity is an important tool for risk estimation; therefore, it has been studied using both conventional histopathological mitotic count and analysis of the Ki67 proliferation index. The histopathological subtype is of utmost importance for assessing disease progression and survival for liposarcoma, as pleomorphic and dedifferentiated subtypes often have an unfavorable evolution, while a well-differentiated liposarcoma/atypical lipomatous tumor clinically behaves like locally aggressive neoplasms. In a previous study that we published, we created an algorithm with prognostic–predictive significance for liposarcoma, the LEMON (Liposarcoma Evaluation Mitosis Origin Necrosis) two-tiered system, integrating histological subtype, mitotic activity, and tumor necrosis. The aims of the present study are to depict the overall survival of patients with liposarcoma stratified by Kaplan–Meier analysis categorized by tumor histological grade and to underscore the clinical utility of the LEMON score in risk stratification segregating indolent (low-risk) from aggressive (high-risk) liposarcomas across histological grades. *Materials and Methods*: We carried out a retrospective multicenter study on 99 patients diagnosed with primary liposarcoma between 2009 and 2023 who were followed up to assess the presence of metastases and their survival period. We performed Kaplan–Meier analysis for overall survival. Proliferative tumor activity was analyzed using conventional histopathological examination and Ki67 immunostaining, and the methods’ sensitivity was compared using Bland–Altman analysis. *Results*: In this respect, tumors with a higher histological grade were associated with worse survival with statistically significant differences in survival between G1 and G3 liposarcomas. Ki67 immunostaining proved to be more sensitive in detecting cellular proliferation compared to histologically observed mitoses. Furthermore, the risk stratification of cases by tumor grade and LEMON score effectively segregates indolent lesions (low risk) from aggressive subtypes (high risk) and may have clinical utility. *Conclusions*: The histopathological examination for liposarcoma subtype, mitotic index, and tumor necrosis is crucial for assessing the risk of progressive disease and the overall survival of patients. This study focuses on describing the prognostic significance of tumor grade, emphasizing proliferative activity evaluation. The clinical utility of a two-tiered system classifying liposarcomas into “low-risk” and “high-risk” lesions can be evaluated by providing an overview of overall survival in relation to histological grade and LEMON risk score. Risk stratification is particularly important in identifying the patients with liposarcoma who may benefit from intensified surveillance or adjuvant therapies.

## 1. Introduction

Liposarcomas are some of the most complex mesenchymal tumors, exhibiting variable clinical evolution and general prognosis [1]. Histopathological subtype, tumor grade, proliferative activity, and tumor necrosis are among the most important prognostic factors in the matter of liposarcoma [2,3,4,5]. Studies available in the scientific literature acknowledge that certain liposarcoma variants have been associated with a higher risk of progressive disease and with reduced survival [1,3,5]. Dedifferentiated and pleomorphic liposarcomas are typically associated with a poor prognosis [6,7,8]. The specific features regarding the clinical evolution of these tumors are correlated with their histopathological and molecular characteristics and, in some cases, with their peculiar location [7,9]. Metastatic liposarcomas often exhibit high-grade morphology and a high mitotic index, highlighting the need to identify clear criteria for risk appraisal in patients diagnosed with these malignancies [5,9,10]. According to the FNCLCC system, mitotic activity is a prognostic tool for every liposarcoma subtype, as cell-cycle progression is significantly modified in malignant tumors [11,12]. Immunohistochemical analysis is an ancillary method successfully used for the diagnosis and prognostic characterization of liposarcomas [13,14,15]. The identification of MDM2, CDK 4, and DDIT3 expression is useful in confirming liposarcomas associated with the subsequent genetic alteration, while analysis of Ki67 expression is useful for determining the mitotic index and tumor grade [11,13,14,15]. Tumor necrosis is also a histopathological factor with prognostic value, as we found that necrosis was present in the majority of high-grade liposarcomas and also has been reported as significant for survival in the scientific literature [16]. In our study, we found that the histopathological subtype, proliferative activity, and tumor necrosis were statistically significant for disease progression and survival in cases of liposarcoma; therefore, we developed a new algorithm for classifying malignant lipomatous tumors into subclasses with prognostic–predictive value to provide a more detailed description of the malignant lesions in each patient and subsequently evaluate potential responses to personalized therapy. Additionally, tumor grade showed prognostic significance in the evaluated patients. In this context, we conducted a Kaplan–Meier analysis of overall survival and a comparative analysis of the statistical significance between our proposed risk score and tumor grade.

### Hypothesis Statement

**Null Hypothesis** **(H_0_):** *Tumor histological grade, mitotic index, tumor necrosis, and the LEMON score have no significant association with overall survival in patients with liposarcoma or atypical lipomatous tumor*.

**Alternative Hypothesis** **(H_1_):** *Tumor histologic grade, mitotic index, tumor necrosis and the LEMON score are significantly associated with overall survival in patients with liposarcoma or atypical lipomatous tumor, with higher grades and LEMON score, predicting worse prognosis*.

## 2. Materials and Methods

### 2.1. Study Participants

The presented survey is the second phase of a retrospective study which included 99 patients diagnosed with primary liposarcoma of the somatic soft tissue, viscera, retroperitoneum and mediastinum in the Clinical Pathology Laboratory of the University Emergency Hospital of Bucharest, Romania and in the Department of Pathology of the Clinical Emergency Hospital of Bucharest, Romania between 2009 and 2023. The cases were collected in the context of a retrospective chart review. At first, 107 cases were identified, but 8 of them were excluded due to lack of follow-up. Statistical analysis was initially performed on 77 patients for whom results on proliferative activity evaluation methods were provided. The study group was later completed and added to a total of 99 patients. The following inclusion criteria for the patients were used: a histopathological diagnosis of primary liposarcoma, imagistic investigations used to assess the presence of secondary tumor determinations after the initial diagnosis and a follow-up of at least two years. The cases were confirmed by immunohistochemical analysis, and next-generation sequencing was also performed in a tertiary diagnostic center for cases with non-specific immunophenotype. We excluded the patients who had previously received neoadjuvant therapy to avoid misinterpretation of post-radiation necrosis as intrinsic tumor necrosis and patients for whom the follow-up period could not be obtained. During the follow-up period, we assessed progression-free survival (PFS) and overall survival (OS).

The study was approved by the Ethics Committee of the University Emergency Hospital of Bucharest by decision nr. 79363/21 December 2023 and by the Ethics Committee of the Clinical Emergency Hospital Romania Bucharest by decision nr. 5210/3 July 2024 and was conducted in accordance with the principles of the Helsinki Declaration. Each patient signed an informed consent form.

Study’s limitations

The limitations of the presented study consist of the following:

- Sample size and distribution:

The number of cases in our study group is limited. This may reduce the statistical power to detect significant differences or subtle variations across the subgroups. This study has a retrospective design and relatively modest sample size of 99 patients. The limitation is reflected in wide confidence intervals observed for certain hazard ratios (HR = 2.43, 95% CI: 1.00–5.92), raising the possibility of type 2 errors and the underpowered detection of significant associations with specific subgroups. Consequently, some prognostic relationships may be underestimated or missed.

- Selection bias:

Another limitation may arise from the exclusion of patients who received neoadjuvant therapy. This criterion may skew the results toward a more inherent native tumor behavior, potentially limiting the generalizability of findings to liposarcoma cases. Additionally, incomplete data on important confounding variables such as patient’s ethnicity and duration or consistency of follow-up may further impact the robustness and applicability of our conclusions.

- Retrospective and cross-sectional design of the study:

The study assesses the Ki67 values at a single time point, while longitudinal follow-up data would be required to correlate with clinical outcomes, such as metastases and survival.

- Heterogeneity within tumor grade:

The observed variability within G2 tumors suggests biological heterogeneity which may not be totally captured by Ki67 alone. Further molecular and genetic analysis can enhance risk stratification.

- Potential interobserver variability:

Assessment of Ki67 by immunohistochemical analysis can be subject to interobserver variability, depending on staining protocols and scoring methods.

### 2.2. Clinical, Histopathological and Immunohistochemical Analysis

The tissue samples were processed by standard histopathological and immunohistochemical methods. Three pathologists (Ana-Maria Ciongariu, Adrian-Vasile Dumitru and Valentin Enache) examined the sections and established the diagnosis. Differences in opinion were settled by consultation with other two pathologists (Mariana Costache and Cosmin Creanga). We used the following immunohistochemical markers for confirmation: CDK 4 (cyclin-dependent kinase-4)—ZR349, MDM2 (murine double minute 2)—SMP14 mouse monoclonal 1:50, Zeta; rabbit monoclonal 1:100, Zeta; S100 protein—4C4.9, mouse monoclonal 1:150, Zeta and p53-DO-7 mouse monoclonal 1:100, Zeta. Proliferative activity within the samples was analyzed using Ki67—MIB 1, mouse monoclonal, Zeta (Zeta Corporation, Monrovia, CA, USA).

Mitotic activity was evaluated by counting mitotic figures in hematoxylin and eosin (H&E)-stained tumor sections under high-power fields (HPF, 400× magnification). In the first phase of the study, the cut-off values of 2 mitotic figures/10 HPF and 5 mitotic figures/10 HPF were chosen, as these were the median values in patients without metastases and in patients with metastases, respectively.

For the purpose of analysis, necrosis was recorded using a binary classification system, as either present or absent, without quantifying the percentage of necrotic area. This approach was chosen to maintain consistency and reproducibility in the assessment across cases.

Clinical data, imaging studies, and surgical management protocols were reviewed and interpreted collaboratively by an orthopedic surgeon and a plastic surgeon (Cătălin Cîrstoiu and Andrei Marin). Data collection and statistical analysis were conducted by Ana-Maria Ciongariu and Șerban-Ștefan Radu.

### 2.3. Data Collection and Analysis

For each patient, we collected the following variables: histological subtype of liposarcoma, mitotic index (measured by conventional histopathological evaluation), Ki67 proliferation marker value, presence of necrosis, and duration of survival post-diagnosis (in months). Data were obtained by investigating the patients’ medical records and retrieval of information from the National Health Insurance House system.

All cases were revised before performing statistical analysis and liposarcoma subtypes; grade and TNM staging were classified according to the 5th edition of The WHO Classification of tumors of the soft tissue and bone and to the guidelines of Fédération Nationale des Centres de Lutte Contre le Cancer (FNCLCC). We thoroughly selected only patients without neoadjuvant therapy in order to investigate the native tumor heterogeneity and avoid the evaluation of treatment-induced cellular changes. Descriptive statistics were provided, including mean, standard deviation (SD), median and range for continuous variables and frequency with percentage.

Continuous variables were analyzed using the Mann–Whitney U test and Student’s *t*-test, while survival analysis was performed using the Kaplan–Meier method. Categorical variables were evaluated with Fisher’s exact test. Relative risks (RRs) were calculated along with 95% confidence intervals (CI). Statistical significance was defined as *p* < 0.05.

Analyses involving the Mann–Whitney U test and Student’s *t*-test were conducted using GraphPad Prism version 10.2.3 (GraphPad Software Inc., San Diego, CA, USA). Survival analysis was carried out using Python version 3.13.3. Kaplan–Meier survival curves and hazard rate estimations were generated using the lifelines library, and graphical representations were produced with matplotlib. Data processing and organization were performed using pandas, and additional descriptive analyses were completed in Microsoft Excel.

### 2.4. Data Availability

The datasets generated and/or analyzed during the current study are not publicly available due to patient confidentiality and institutional restrictions. Access to the data was granted exclusively following approval from the Ethics Committee as well as the Hospital Management and Heads of the Pathology Departments/Laboratories involved.

## 3. Results

### 3.1. Tumor Grade

Histologic grade was statistically significant for survival, as high-grade tumors, particularly G3 liposarcoma, were associated with worse overall survival. The median value for survival in patients with grade 1 tumors was 15.80 (interval 0.23–180.3; range: 178.8) and 16.20 (0.00–104.27; range: 104.27) for patients with G3 tumors, respectively, with *p* values G1 vs. G3 = 0.0013. The mean survival for patients with G1 tumors was 40.70 (interval 0.23–180.3), while it was 29.87 for G2 (interval 0.10–134.56; range: 134.46) and 23.66 for G3 (interval 0.00–104.27).

At 1 year, survival rates were estimated at 93% (G1), 90% (G2), and 73% (G3). By 3 years, survival declined to 89% (G1), 80% (G2), and 50% (G3). By year 5, survival further diverged, with G1 and G2 remaining stable (89% and 80%, respectively), while G3 declined sharply to 35%.

Pairwise log-rank testing demonstrated statistically significant differences in survival between G1 and G3 (*p* = 0.0013), and between G2 and G3 (*p* = 0.0237), while no significant difference was observed between G1 and G2 (*p* = 0.4513).

**Figure 1:**
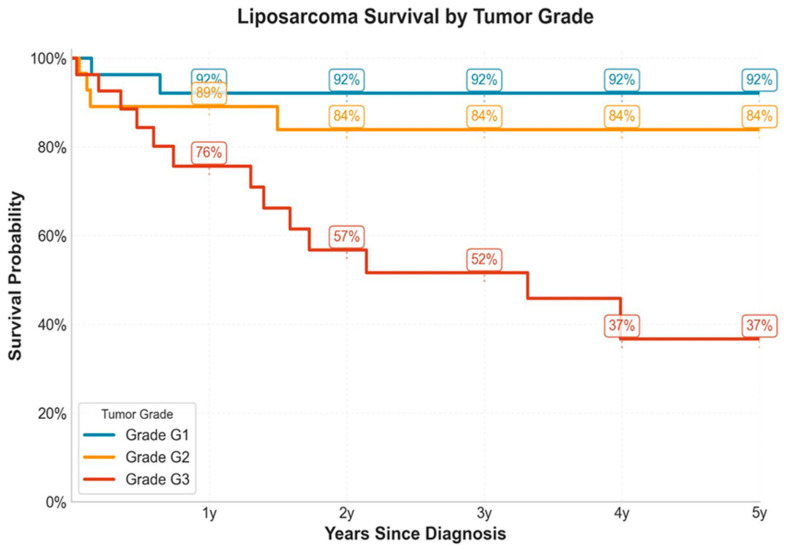
Kaplan–Meier survival curves stratified by tumor grade in liposarcoma patients.

These findings underscore the prognostic significance of histologic grade in liposarcoma with higher-grade tumors (particularly G3) associated with significantly worse overall survival (Figure 1).

**Figure 2:**
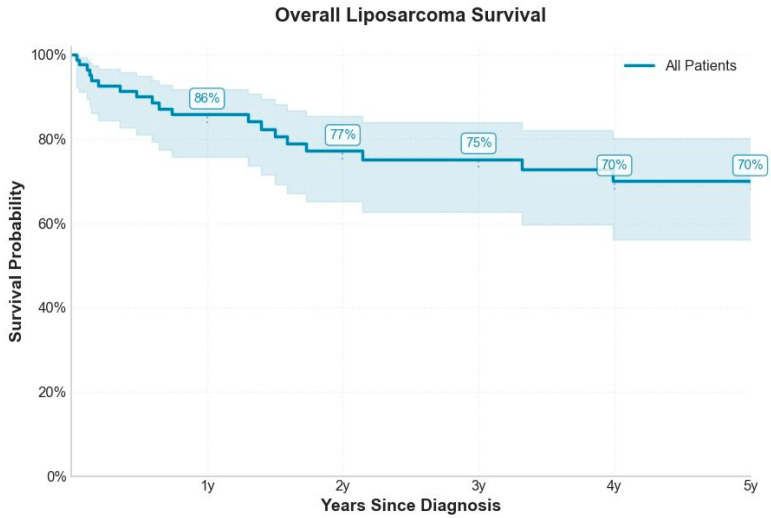
Kaplan–Meier analysis for overall survival in patients with liposarcoma.

This Kaplan–Meier survival analysis depicts overall survival for 99 patients with liposarcoma, which was stratified by tumor histologic grade (G1, G2, G3) over a five-year follow-up period—see Figure 2.

Grade G1 (n = 37) is shown in blue and is associated with the most favorable prognosis with a stable survival trajectory and a 5-year survival rate of approximately 89%. Grade G2 (n = 32), represented in orange, exhibits intermediate survival, with a modest decline in the first two years and a plateau thereafter; the 5-year survival rate is approximately 80%. Grade G3 (n = 30), illustrated in red, shows a substantially poorer prognosis, with a steep decline in survival over time, culminating in a 5-year survival rate of approximately 35%.

### 3.2. Proliferative Activity

**Figure 3:**
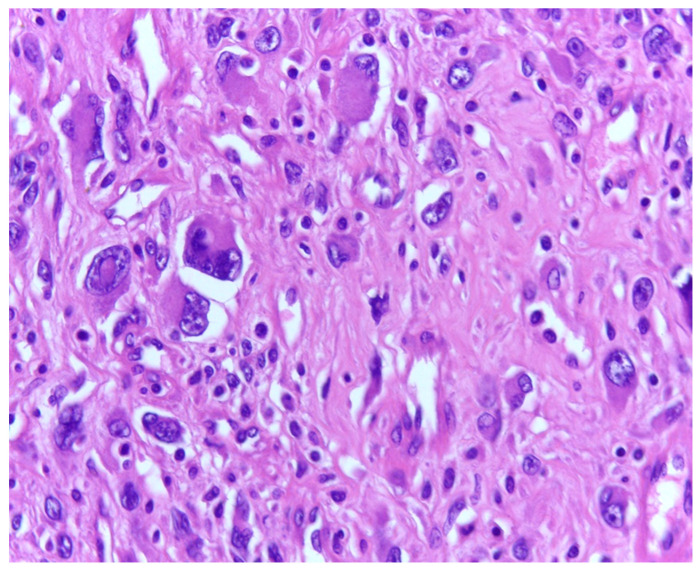
Marked pleomorphism and mitotic activity in a dedifferentiated liposarcoma; H.E., 200×.

As we earlier mentioned, the first phase of the study implied the identification of histopathological characteristics with prognostic significance for survival and disease progression in the matter of liposarcoma—see Figure 3. In our group of patients, mitotic activity was statistically significant. A subgroup of 77 patients was analyzed to assess the significance of proliferative activity, which was studied using two methods. First of all, the mitotic index was determined by counting the number of mitotic figures/10 high power-fields (HPFs). The mean mitotic count in tumors of the patients who survived during follow-up was 3.562/10 HPF (median = 2, range: 1–23), while that in those of patients who died was 8.65/10 HPF (median = 5, range 1–25). A higher mitotic index is also significantly associated with decreased OS (Mann–Whitney test, *p* = 0.0011). Analysis of immunohistochemical testing of Ki67 expression was performed for 75 patients with the following results: the mean value of Ki67 for patients who died during follow-up was 8.7780952 (interval 0.95–24.6; range 23.65), while the mean value for patients who survived was 3.4234545 (interval 0.93–22.6; range 21.67).

The results of the immunohistochemical testing of proliferative activity were compared with conventional evaluation using standard microscopic slides in order to detect any potential differences in the sensitivity of the two methods. From the analyses performed, we found that in the case of patients who had died, the median Ki67 value was 4.88 (minimum = 0.95; maximum = 24.6), while in patients who survived, the median mitotic index was 1.95 (minimum = 0.93; maximum = 22.6).

The comparison between immunohistochemical testing and conventional histological evaluation reveals important insights into their perspective sensitivity in assessing tumor proliferative activity. The use of Ki67, a well-established marker for cellular proliferation, provides a better quantitative and potentially more sensitive method compared to the mitotic index derived from standards microscopy.

The data obtained indicate a clear distinction in proliferative activity between patients who died and those who survived. Specifically, the median Ki67 index in deceased patients was significantly higher compared to the median mitotic index in survivors. This difference suggests a possible correlation between higher proliferative activity and poor prognosis, emphasizing the prognostic value of this proliferation marker in clinical assessment.

Moreover, the range of Ki67 values in deceased patients overlaps with that in surviving patients, indicating that while there is a general trend, the results obtained in individual cases may vary substantially. This aspect highlights the need for a multifactorial approach to prognosis in a way that includes both quantitative markers like Ki67 and traditional pathological evaluation. These findings underscored the potential of immunohistochemical techniques to enhance the accuracy of the prognostic evaluation of patients with liposarcoma. However, it is important to consider the methodological differences when interpreting such data. Further studies are warranted to standardize the use of Ki67 and integrate it with existing diagnostic protocols to improve patient stratification and therapeutic strategy development.

**Figure 4:**
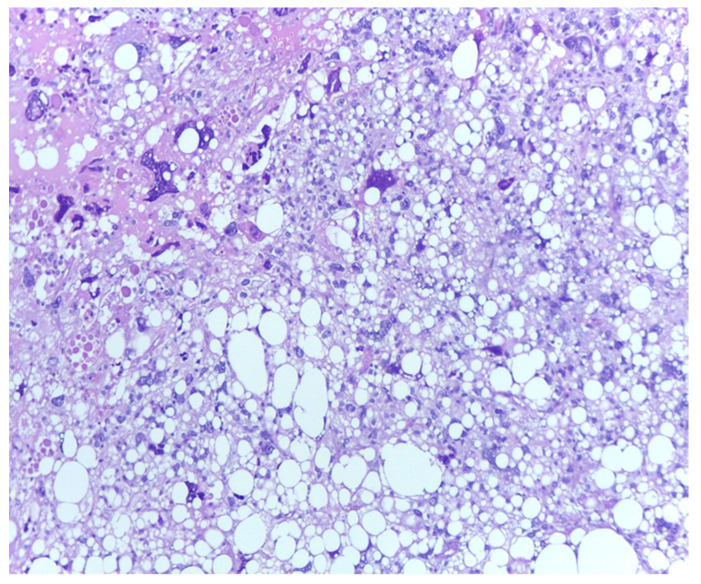
Atypical mitotic figures and marked hyperchromasia in a pleomorphic liposarcoma, H.E., 200×.

To enhance our understanding of the accurate assessment of proliferative activity in liposarcoma, we evaluated the agreement between the mitotic index and the Ki67 marker in measuring tumor cell proliferation. Accordingly, we performed a Bland–Altman analysis to assess the agreement between the two methods. The plot showed a mean difference slightly below zero, suggesting that Ki67 values tend to be higher compared to the number of mitoses. Most points fell within the 95% limits of agreement with a relatively symmetrical distribution around the mean. This result indicates that Ki67 may be a more sensitive marker of cellular proliferation, capturing a broader range of cells in the cell cycle compared to histologically observed mitoses. The observed differences between the two parameters support the complementary use of Ki67 in the prognostic evaluation of patients with liposarcoma—see Figure 4.

**Figure 5:**
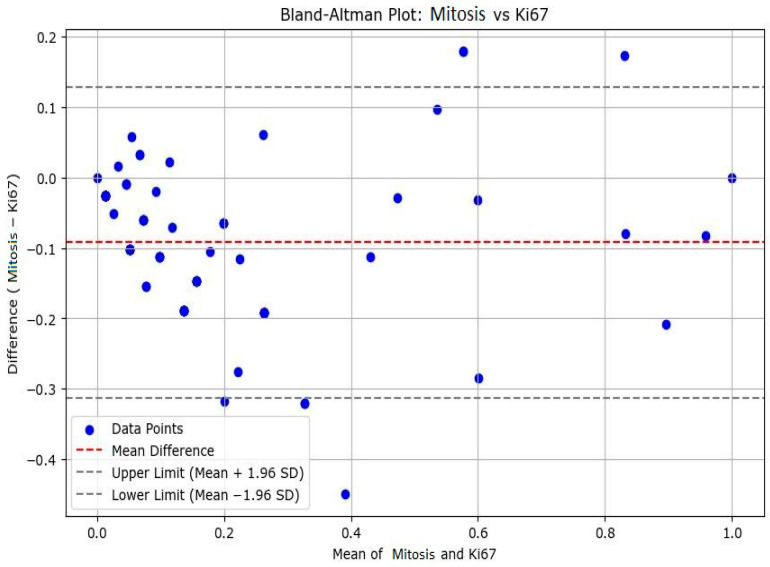
Bland–Altman analysis for mitotic activity and Ki67.

**Figure 6:**
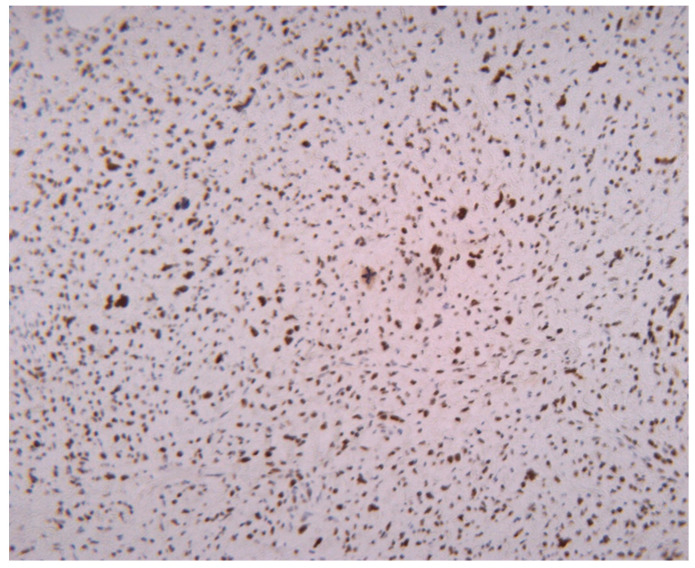
Mitotic activity in a dedifferentiated liposarcoma, P53 mutant immunophenotype with hyperexpression; p53, 100×.

**Figure 7:**
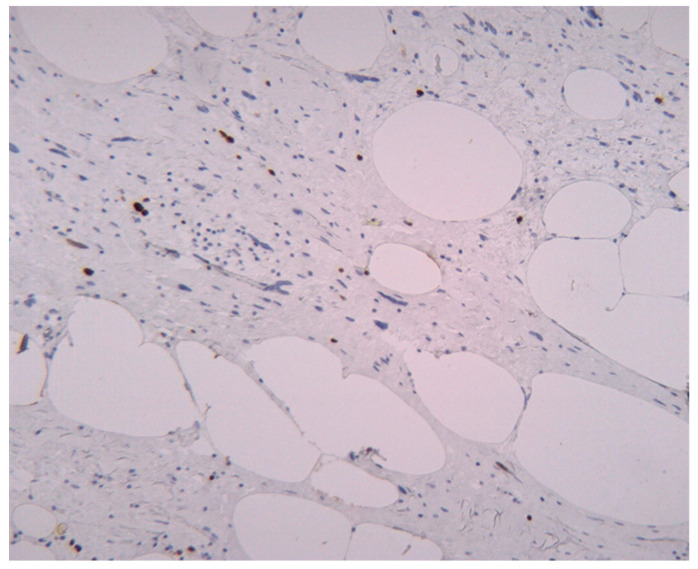
Low proliferative activity in an atypical lipomatous tumor. Ki67 marker proliferation marker expressed in 5% of the tumor cells; Ki67, 200×.

**Figure 8:**
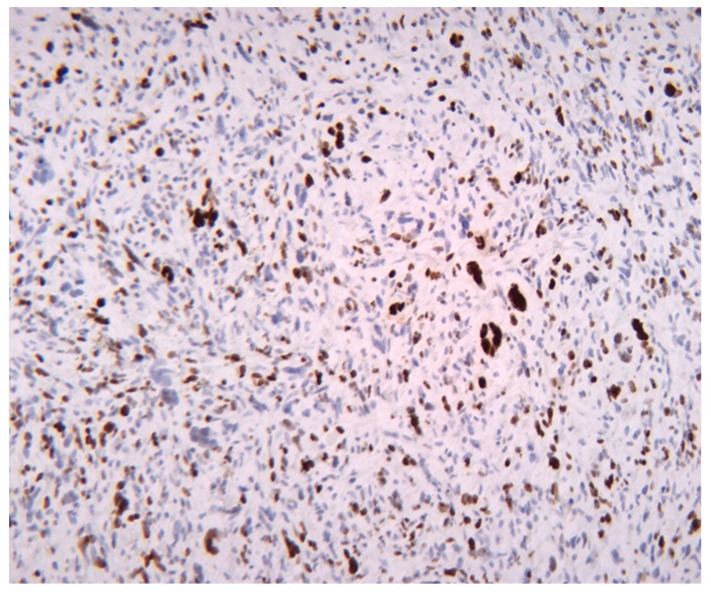
High proliferative activity in a pleomorphic liposarcoma with Ki67 expressed in 60% of the tumor cells; Ki67, 200×.

The presented findings from the Bland–Altman analysis provide meaningful insight into the relationship between Ki67 immunohistochemical analysis and mitotic index evaluation for liposarcoma—see Figure 5. The observation of a mean difference slightly below 0 suggests that Ki67 values generally exceed the number of mitosis identified histologically. These trends indicate that Ki67 may detect proliferative activity not captured by mitotic counts alone, as it can detect cells in a broader range of the cell cycle, including G1, S or G2, rather than being restricted to the relatively short mitotic phase. These symmetrical distribution of data points within the 95% limits of agreement reinforces the reliability of the comparison by indicating no substantial systematic bias across the range of measurements. However, the consistent evaluation of Ki67 values implies that this may be a more sensitive or inclusive marker of tumor cell proliferation. These findings show a potential clinical value of Ki67 as a complementary tool in the assessment of proliferative activity in liposarcoma—see Figure 6, Figure 7 and Figure 8. However, while the mitotic index remains the cornerstone of histopathological evaluation, the integration of Ki67 can enhance prognostic accuracy by identifying the tumors with high proliferative activity that may otherwise be underestimated by conventional methods alone. The complementary use of both markers could therefore inform risk stratification and contribute to the formulation of more individualized therapeutic approaches.

### 3.3. Prognostic Value of the LEMON Score in Evaluating Overall Survival in Patients with Malignant Lipomatous Tumors

As we previously mentioned, we conducted and published an original study analyzing the statistical significance of histopathological characteristics of liposarcoma for patients’ risk for disease progression and death. We developed a novel prognostic algorithm, designated as the LEMON score (Liposarcoma Evaluation Mitosis Origin Necrosis), which integrates the histologic subtype, mitotic index and the presence of tumor necrosis for risk stratification. By systematically integrating these variables, the LEMON score enables the stratification of patients according to their estimated risk. It provides a two-tiered classification system, encompassing both low-risk and high-risk lesions, thereby offering an appropriate classification for liposarcoma, aiming at a personalized therapeutic approach. The LEMON score is a prognostic tool used to assess the risk of progressive disease and death in liposarcomas, integrating three key parameters: histologic subtype, mitotic index and the presence of tumor necrosis. Each criterion is assigned a point value based on its prognostic severity with higher scores indicating a potentially more aggressive tumor behavior. The thresholds for mitotic activity were established based on observed differences between patients with and without metastases. Tumors with a score between 3 and 5 are classified as low risk, while tumors with a score of 6 to 8 fall into the high-risk category. By carrying out statistical analysis using Fisher’s exact test, we demonstrated a significant association between high-risk classification based on the LEMON score and the presence of progressive disease (RR = 8.853; 95% CI: 2.479–33.52; *p* = 0.0001).

In the subsequent phase of the study, we evaluated the overall survival in patients with liposarcoma based on the LEMON score, which incorporates mitotic index, histologic subtype, and tumor necrosis. Patients with lower scores (3–5), corresponding to the low-risk category, demonstrated a consistently high survival rate over time, maintaining an 80% survival at five years. In contrast, patients classified as high-risk (scores 6–8) experienced a marked decline in survival, reaching only 54% at the five-year mark. This difference was statistically significant with a *p*-value of 0.0424—see Table 1. The findings of this study highlight the prognostic utility of the LEMON score in stratifying overall survival among patients with liposarcoma. The marked disparity in five-year survival between low-risk and high-risk groups underlines the system’s effectiveness in distinguishing between prognostically distinct patients populations. The statistical significance of this difference (*p* = 0.0424) reinforces the robustness of the LEMON score as a predictive measure. A clinical perspective can provide a better understanding of the usefulness of the score and its potential influence on decision-making and the ability to tailor a personalized approach. For example, patients with high-risk tumors may benefit from more aggressive treatment regimens or closer surveillance, whereas patients with low-grade lesions might avoid exposure to overtreatment. Moreover, the consistent survival in the low-risk group over time suggests that the LEMON score could potentially identify patients with more indolent forms of tumors who may have favorable evolution with standard treatment alone. In this respect, future prospective studies and external validation in larger cohorts are required to further refine the LEMON system and confirm its applicability across a diverse patient population.

**Figure 9:**
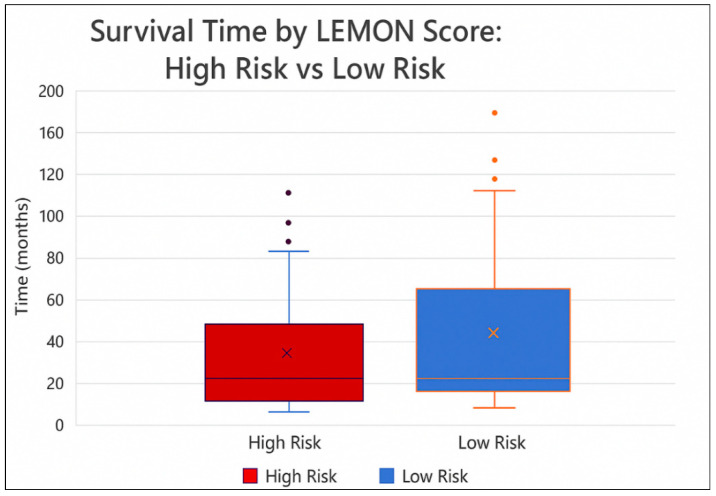
Survival time by LEMON score.

Descriptive statistical analyses of the LEMON score in relation to survival were performed on a cohort of 99 patients. Among the 99 patients, 51 belonged to the high-risk group and 48 belonged to the low-risk group. The mean survival time for patients in the high-risk group was 26.49 months (range = 0–129.27; SD = 31.16; 95% CI: 17.94–35.04) with a median survival of 17.48 months. In the low-risk group, the mean survival was 37.93 months (range = 0.23–180.03; SD = 43.37; 95% CI: 25.65–50.20), and the median survival was 15.26 months. The apparent inconsistency between the mean and median values—particularly the lower mean survival in the low-risk group despite a higher median—is attributable to data skewness and increased variability. Specifically, the low-risk group showed a higher standard deviation (43.37 vs. 31.16) and a greater positive skew (1.87 vs. 2.74), indicating that survival times were more dispersed and included early deaths or early censoring events that pulled the mean downward. This highlights the importance of using both central tendency measures and considering the distribution shape when interpreting survival data. Despite this, the LEMON score remains a valid risk stratification tool, as shown by the statistically significant hazard ratio from the Cox regression analysis. Patients in the high-risk group (LEMON score 6–8) showed a significantly increased risk of death compared to those in the low-risk group (LEMON score 3–5), with a hazard ratio (HR) of 2.43 (95% CI: 1.00–5.92, *p* = 0.05), according to Cox regression analysis—see Figure 9.

**Figure 10:**
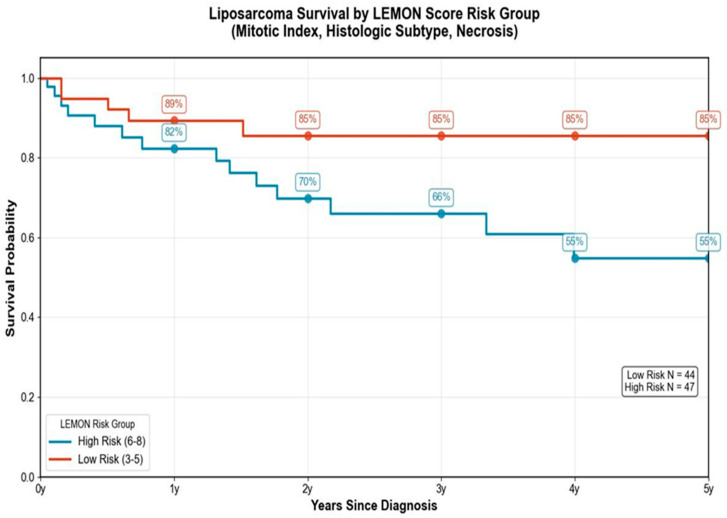
Liposarcoma survival by LEMON score.

The descriptive statistical evaluation of survival in relation to the LEMON score provides valuable insight into its prognostic capacity. The observed differences in the mean and median survival between the two risk groups described underscore the challenges inherent in interpreting survival data in the presence of skewed distributions. Specifically, patients diagnosed with low-risk tumors demonstrated greater variability and positive skewness, likely due to a small number of early deaths or censored observations, which lowered the mean in spite of a higher median. This aspect emphasizes the importance of using multiple statistical measures to fully capture the survival profile of patient subgroups. To go on, the survival disparity between groups was supported by inferential analysis. The Cox regression model identified a significantly elevated hazard of death in the high-risk group (HR = 2.43), reinforcing the LEMON score’s predictive utility. Although the confidence interval borders on the threshold of significance, the findings remain clinically meaningful and suggest that the score effectively captures biologically relevant features of tumor aggressiveness, such as mitotic activity and necrosis—see Figure 10.

The Kaplan–Meier survival curve provides an overview of the overall survival of all patients included in the study. This analysis offers a comprehensive view of the overall survival trajectory in the study cohort. In the present analysis, the estimated survival rate at one year after diagnosis was 86%, gradually declining to 75% at three years and reaching 68% at five years, outlining a moderately decreasing trajectory. The observed survival rates indicate a relatively favorable prognosis for patients with liposarcoma. Notably, most deaths occurred within the first two years of follow-up, indicating this interval as the period of highest risk for patients. The sharpest decline in survival occurred within the first two years post-diagnosis, identifying this period as the most critical for patient outcomes. This pattern underscores the need for vigilant monitoring and potentially intensified therapeutic interventions during early follow-up. Although the overall prognosis appears moderate to good, these findings highlight the time-dependent dynamics of risk and the importance of early risk stratification tools such as the LEMON score in guiding surveillance and treatment strategies.

**Figure 11:**
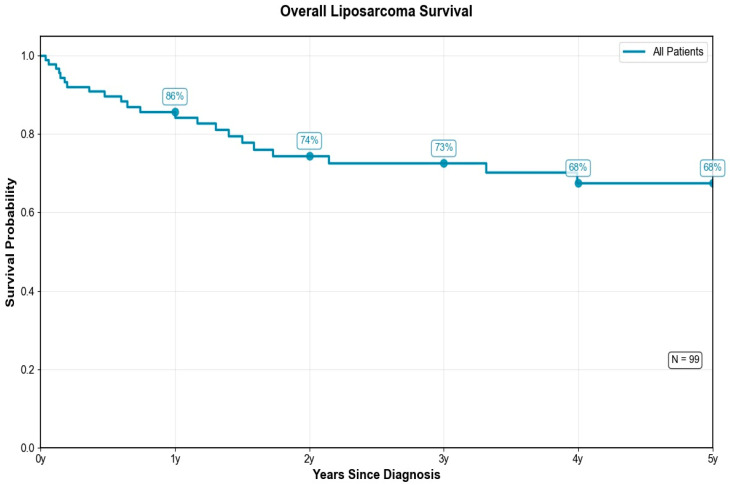
Kaplan–Meier analysis of overall survival in patients with liposarcoma included in the study.

Overall, the survival rate appears relatively favorable for this pathology, but it underscores the importance of close monitoring during the early years following diagnosis—see Figure 11.

### 3.4. Distribution of Liposarcoma Cases by Tumor Grade and LEMON Risk Score

In order to gain further knowledge about risk assessment in liposarcoma, we evaluated the relationship between histological tumor grade (G1–G3) and LEMON score-based risk stratification. By analyzing how tumors of different grades distribute across the low-risk and high-risk LEMON categories, the study sought to validate the LEMON score’s correlation with established tumor grading, particularly the FNCLCC system (which includes mitotic index, necrosis, and differentiation). Furthermore, we investigated whether the LEMON score can effectively capture the biological aggressiveness of various histological subtypes of liposarcoma. We also highlight the heterogeneity within intermediate-grade (G2) tumors, which may present with a broader spectrum of prognostic features, leading to both low- and high-risk classifications.

**Figure 12:**
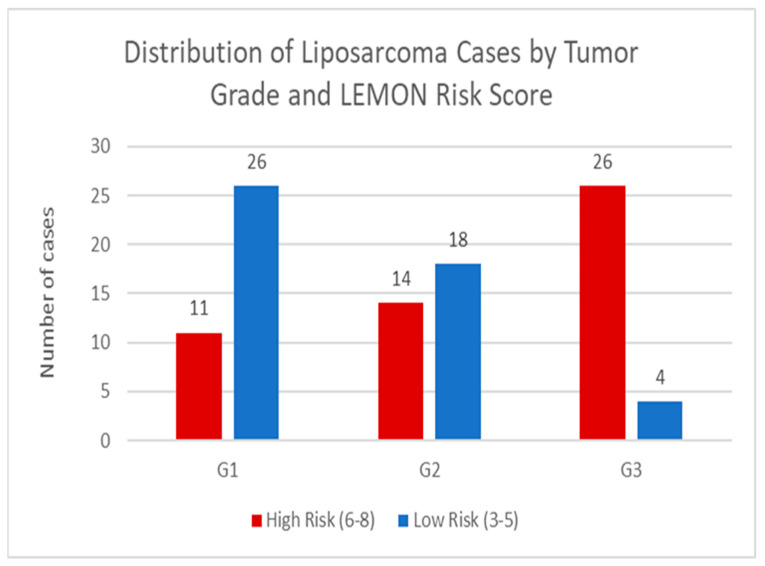
Distribution of liposarcoma cases by tumor grade and LEMON score.

The presented graph illustrates the distribution of liposarcoma cases stratified by tumor grade (G1, G2, G3) and the LEMON (Liposarcoma Evaluation Mitosis Origin Necrosis) risk score, which categorizes tumors into low-risk (3–5 points) and high-risk (6–8 points) groups. The LEMON score integrates three critical histopathological parameters: histological subtype, mitotic index, and the presence of tumor necrosis. G1 tumors: The majority (26 cases) were classified as low risk with 11 cases falling into the high-risk category. This aligns with the expected biological behavior of well-differentiated liposarcomas, which typically exhibit low mitotic activity and an absence of necrosis. G2 tumors: A significant proportion (18 cases) were low risk, while a smaller subset (14 cases) transitioned to high risk. This reflects the heterogeneity of intermediate-grade tumors, where features such as increased mitotic figures or necrosis may elevate the LEMON score. G3 tumors: Almost all cases (26) were high risk, which was consistent with the aggressive nature of dedifferentiated, pleomorphic, or high-grade myxoid liposarcomas, leaving just 4 cases in the low-risk group. These tumors often demonstrate high mitotic indices (≥5/10 HPF) and necrosis, contributing to their poor prognostic classification—see Figure 12.

The graph underscores the clinical utility of the LEMON score in risk stratification, effectively segregating indolent (low-risk) from aggressive (high-risk) liposarcomas across histological grades. This dichotomy supports its potential integration into therapeutic decision making, particularly in identifying patients who may benefit from intensified surveillance or adjuvant therapies. Moreover, the graphical distribution of liposarcoma cases, stratified by tumor grade and LEMON risk score, provides compelling evidence for the potential clinical relevance of this composite scoring system. By integrating histological subtype, mitotic index and tumor necrosis, otherwise considered critical indicators of aggressiveness in sarcomas, the LEMON score offers a comprehensive framework for prognostic evaluation that provides additional value over conventional grading. Furthermore, the observed patterns across tumor grades reinforce the biological plausibility of the LEMON score. In G1 tumors, the predominance of low-risk profiles aligns with the characteristic indolent nature of well-differentiated liposarcomas, which rarely exhibit high mitotic activity or necrosis. Conversely, the mixed-risk distribution seen in G2 tumors highlights the inherent heterogeneity of intermediate-grade lesions. The classification of some G2 lesions into the high-risk category likely indicates subtle but clinically significant histopathological features such as increased proliferation or early necrotic transformation that may not be fully captured by grade alone. Notably, the near universal classification of G3 tumors as high risk supports the sensitivity of the LEMON score in identifying biologically aggressive liposarcomas such as dedifferentiated and pleomorphic variants. These entities are characterized by high mitotic rates and necrotic areas—both of which directly elevate the LEMON score and correlate with poor clinical outcomes. Importantly, the stratification achieved through the LEMON score has direct implication for patient management. By distinguishing between low and high-risk tumors with each histological grade, the score enhances prognostic accuracy and may inform more individualized therapeutic decision making. For instance, patients with high-risk tumors, regardless of grade, could be candidates for intensified postoperative surveillance, adjuvant radiotherapy or systemic therapies. In contrast, those diagnosed as low risk could potentially be spared from overtreatment, reducing exposure to unnecessary therapeutic burden. Overall, the obtained data support the LEMON score as a valuable adjunct to the traditional grading system, offering refined prognostic stratification and the potential to guide risk adaptive clinical management in patients with liposarcoma.

### 3.5. Distribution of Liposarcoma Tumor Grades by Ki67 Proliferation Index

**Figure 13:**
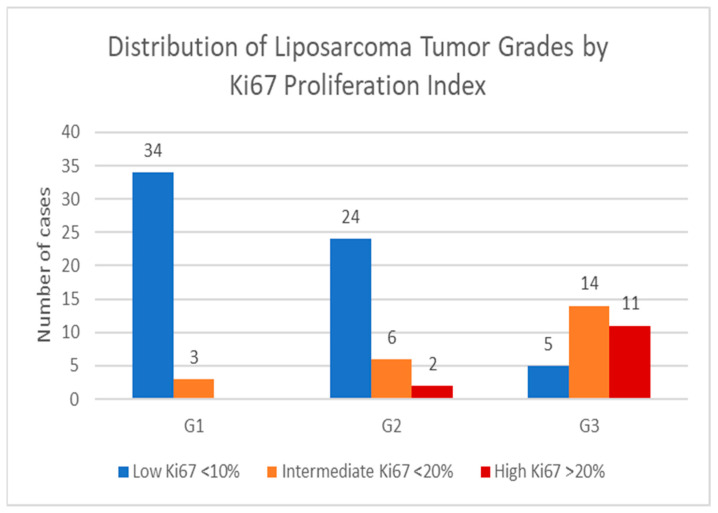
Distribution of liposarcoma tumor grades by Ki67 proliferation index.

This analysis was conducted to evaluate the relationship between liposarcoma tumor grade (G1–G3) and the Ki67 proliferation index, which is a marker of cellular proliferation. By categorizing tumors into low (<10%), intermediate (<20%), and high (>20%) Ki67 expression groups, the study aimed to assess how proliferation activity correlates with histological grade, providing insight into tumor aggressiveness. Additionally, the study examined whether higher-grade tumors are associated with increased Ki67 expression, which could support the role of Ki67 as a potential prognostic biomarker—see Figure 13.

The presented graph illustrates the distribution of liposarcoma cases stratified by tumor grade (G1, G2, G3) and Ki67 proliferation index, which categorizes tumors into low (<10%), intermediate (10–20%), and high (>20%) proliferative activity groups. The Ki67 index serves as a reliable marker of cellular proliferation with higher values indicating more aggressive tumor biology.

Key observations from the graph are outlined below. G1 tumors: The vast majority (34 cases, 92%) exhibited low Ki67 expression with a small subset (3 cases, 8%) showing intermediate proliferation. No G1 tumors demonstrated high proliferative activity. This pattern aligns with the expected indolent behavior of well-differentiated liposarcomas, which typically display minimal cellular turnover. G2 tumors: These intermediate-grade tumors showed greater biological variability with 24 cases (75%) maintaining low proliferation, 6 cases (19%) exhibiting intermediate activity, and 2 cases (6%) displaying high Ki67 levels. This spectrum reflects the transitional nature of G2 tumors, where some cases may acquire more aggressive proliferative features. G3 tumors: Poorly differentiated tumors demonstrated markedly elevated proliferation with only five cases (17%) retaining low Ki67 expression. The majority showed either intermediate (14 cases, 47%) or high (11 cases, 37%) proliferative activity, which was consistent with the known aggressive behavior of high-grade liposarcomas.

This analysis highlights the strong correlation between tumor grade and proliferative activity with Ki67 serving as a valuable adjunct to histological assessment. The clear progression from low proliferation in G1 tumors to increasingly higher proliferation in G3 tumors underscores the utility of Ki67 immunohistochemistry in risk stratification.

By analyzing the Ki67 proliferation index in relation to liposarcoma tumor grade, we provide significant insight into the biological behavior and aggressiveness of these soft tissue tumors. This stratification of tumors into low, intermediate and high-proliferative activity groups based on Ki67 expression facilitates a detailed understanding of cellular turnover across different histologic tumor grades. The presented data revealed clear and expected gradient in proliferative activity, closely corresponding with tumor grade. In G1 tumors, the predominance of low Ki67 expression reflects their indolent nature, which is histologically emphasized by minimal cellular proliferation. Additionally, the absence of high proliferative activity in this group supports the generally favorable prognosis associated with these tumors. Intermediate-grade (G2) tumors show greater heterogeneity in proliferation rates, with the majority of them exhibiting low Ki67 levels, yet a notable fraction displaying intermediate to high proliferation rates. This variability suggests a transitional biological nature for the two tumors, which can possess features of both low- and high-grade lesions. Such heterogeneity may complicate prognostic evaluation based on histological grade alone, thereby highlighting the potential clinical value of integrating Ki67 assessments for more precise risk stratification. High-grade G3 tumors display significantly elevated proliferative activity with the majority showing intermediate or high Ki67 expression. This finding is consistent with the aggressive clinical course typically observed in poorly differentiated liposarcoma such as dedifferentiated or pleomorphic subtypes. These variants, typically associated with increased cellular turnover and poorer outcomes, suggest a correlation between higher Ki67 values and G3 tumors, reinforcing this immunohistochemical marker’s prognostic significance. In summary, this study affirms the strong association between histological grade and cellar proliferation, as measured by Ki67 immunohistochemistry and importantly provides Ki67 as valuable additional information beyond conventional grading.

## 4. Discussion

Considering the marked heterogeneity of malignant lipomatous tumors, several studies have been conducted, aiming to clarify the histopathological and molecular factors related to unfavorable prognosis in liposarcoma [17]. In our cohort, the one-year survival rate for patients with liposarcoma was 86%, decreasing to 75% at three years and 68% at five years, indicating a moderately declining trend. Notably, most deaths occurred within the first two years of follow-up, identifying this interval as the period of highest risk. While overall survival appears relatively favorable for this pathology; however, it highlights the importance of close monitoring during the early years following diagnosis. Consequently, we conducted an in-depth analysis of the histological and immunohistochemical features with potential impact on overall survival. To further elucidate the determinants of clinical outcome, we evaluated key pathological variables, including tumor grade, mitotic activity, proliferative index Ki67, and the presence of necrosis. These parameters were analyzed both individually and in combination, using the integrative prognostic model that we elaborated—the LEMON score. This comprehensive approach enabled the identification of clinically relevant risk groups and underscored the value of histopathological risk stratification in shaping precise diagnosis and tailored follow-up and therapies.

First, based on the findings of our study, FNCLCC guidelines, and the scientific literature, the histologic grading of tumors serves as an important indicator for predicting survival outcomes in patients with liposarcoma [17]. High-grade (particularly G3) sarcomas) have been associated, in most cases, with progressive disease and reduced survival [17,18]. For instance, dedifferentiated liposarcoma was frequently diagnosed in patients with poor prognosis, and researchers have acknowledged this peculiarity is related to MDM2 and HMGA2 expression [17]. Additionally, some surveys highlight the importance of accurate tumor grade evaluation in guiding personalized and potentially targeted therapy [19,20]. In this context, Nyström et al. conducted a study upon tumor-infiltrating immune cells in high-grade sarcomas, aiming to investigate the susceptibility of these malignancies to immunotherapeutic strategies [21]. Researchers noted that tumor grade correlated with CD 163-positive macrophages, which tended to predominate within the tumor microenvironment in liposarcoma and leiomyosarcoma [21]. The same study confirmed that tumor-infiltrating macrophages and tumor necrosis were significantly associated with an increased risk of disease progression, and it suggested that immunomodulatory medication may improve survival for high-grade sarcomas [21]. Ongoing studies in surgical oncology are evaluating the potential benefits of neoadjuvant therapy for high-grade retroperitoneal sarcomas [20]. Although immunohistochemical and molecular analysis are important for the diagnosis and evaluation of liposarcoma, histopathological examination remains a reliable method for tumor grade assessment [18,22]. Suster et al. observed that in a cohort of patients with mediastinal liposarcomas, the mean survival of those with high-grade liposarcomas was significantly shorter compared to patients with low-grade lesions [22]. The upfront imaging-based characterization of sarcomas has also been studied with an attempt to use a CT-based radiomics classification for predicting the histological type and grade of retroperitoneal liposarcoma [23]. This trial emphasizes the importance of tumor grade assessment for malignant adipocytic tumors [23]. In our study, histologic grade emerged as a significant prognostic factor for overall survival with high-grade tumors—particularly grade 3 (G3) liposarcomas—associated with markedly poorer outcomes. Patients with low-grade tumors demonstrated longer survival, while those with intermediate-grade tumors showed intermediate outcomes. The analysis of survival data revealed a gradual decrease in survival rates corresponding to higher tumor grade. Individuals diagnosed with G3 tumors exhibited significantly reduced survival probabilities at 1, 3, and 5 years compared to those with G1 and G2 tumors whose outcomes remained relatively stable over time. These results highlight the prognostic relevance of tumor grade and support its role in risk stratification and clinical decision making. However, tumor grading and prognostic evaluation are complex processes that may require the use of ancillary diagnostic methods [24,25,26,27]. The FNCLCC staging system identifies mitotic rate as a critical prognostic factor across different sarcoma types. In the matter of liposarcoma, this parameter offers essential insights for diagnosis, prognosis and treatment planning [11,18,28,29,30,31]. In some studies, researchers noted a shorter survival and adverse events in patients with a high mitotic index [11,18,28,29,31]. In malignant tumor proliferations, the mitotic cell cycle is dysregulated, as neoplastic cells evade normal mechanisms that control cell division [12]. In high-grade tumor proliferations like dedifferentiated liposarcoma, neoplastic growth is propelled by Rb 1 protein inactivation and mediated by cyclin D-dependent kinases, specifically CDK4 and CDK6 [12]. These molecular mechanisms have formed the basis for studies investigating the efficacy of selective CDK4/6 inhibitors in patients with liposarcoma [12]. Given the importance of intratumoral proliferative activity, it should be assessed using the most appropriate available method [11,16,18]. The evaluation of Ki67 expression using immunohistochemistry serves as an effective ancillary method to measure proliferative activity and can facilitate tumor grading [11,16,18]. For instance, Machado et al. propose that the integration of Ki67 proliferation index evaluation with tumor necrosis assessment enhances the stratification of liposarcomas into low-grade and high-grade categories [32]. They introduced a comprehensive risk assessment model for soft tissue sarcomas, integrating elements from FNCLCC—the CB system, Ki67 expression and the presence of tumor necrosis [32]. Their findings demonstrated that Ki67 is a reliable marker for accurately identifying high-grade tumors [32]. In our study, we compared Ki67 immunohistochemical staining with conventional mitotic index evaluation to investigate proliferative activity in liposarcomas. Ki67 levels were generally higher in patients with poorer outcomes, indicating its potential prognostic value. Bland–Altman analysis showed good agreement between the two methods, although Ki67 appeared more sensitive by detecting a broader spectrum of proliferating cells. These findings support the complementary use of Ki67 alongside mitotic counts in prognostic evaluations. To further elucidate the prognostic value of Ki67, we accomplished another analysis, upon its relationship with liposarcoma tumor grade (G1–G3). Our findings demonstrated a strong correlation between tumor grade and proliferative activity with Ki67 providing valuable complementary information to histological evaluation. The progressive increase in Ki67 expression from low-grade to high-grade tumors underscores its utility in risk stratification. After performing a review of the scientific literature, we highlight the importance of Ki67 evaluation in comparison to other immunohistochemical stains with potential prognostic value [33]. In addition to our findings, a growing body of literature supports the integration of Ki67 into prognostic algorithms. For instance, Takazawa et al. compared the utility of phosphohistone H3 immunohistochemistry as a potential biomarker, in comparison with Ki67, calling attention to their respective prognostic role [33]. Moreover, the Ki67 marker can be useful for the diagnosis of malignant lipomatous tumors in cases with atypical morphology [34]. Sun et al. also demonstrated the prognostic significance of Ki67 labeling in a cohort of 124 patients and underlined the importance of other prognostic pathological factors, such as tumor necrosis, histopathological subtype and local recurrence [35]. Collectively, these findings reinforce the central role of histologic grading proliferative indices in liposarcoma management. Additionally, they advocate for a multidimensional approach that incorporates morphology, immunohistochemistry and molecular insights to guide diagnosis and prognostic evaluation in the matter of liposarcoma.

To improve prognostic validity, we created the LEMON score—a novel risk stratification tool that combines histologic subtype, mitotic index and tumor necrosis. Building upon prior findings, this score was designed to estimate the risk of disease progression and mortality. Our results demonstrated a statistically significant association between the LEMON score and overall survival with high-risk classifications correlating with increased metastatic potential and reduced survival. The score showed predictive value in both disease progression and long-term outcomes, as confirmed by statistical testing. Notably, it can be applied rapidly during routine histopathological evaluation without incurring additional costs or requiring advanced subspecialty expertise.

Further analysis assessed the concordance between LEMON-based risk groups and established histologic grades (G1–G3), particularly the FNCLCC system. This comparison confirmed that the LEMON score reflects tumor aggressiveness and provides additional prognostic stratification. Intermediate-grade (G2) tumors exhibited notable heterogeneity with cases distributed across both low- and high-risk groups—highlighting the score’s ability to capture subtle differences in tumor biology. These findings support the utility of the LEMON score as a practical, reproducible tool for risk assessment in liposarcoma and brings out the need for its validation in larger, independent cohorts. The rationale for this approach stems from the widely variable clinical behavior of liposarcoma, driven by its underlying histopathological diversity.

Although histopathological and immunohistochemical analyses remain essential in the diagnosis and management of liposarcoma, molecular and genetic analyses are becoming essential in select instances, especially for tumors showing atypical features or those occurring in uncommon sites [15,36,37,38]. These malignancies may mimic other mesenchymal or even benign proliferations, necessitating ancillary testing for accurate classification [37,38]. For instance, the distinction between atypical lipomatous tumors and spindle cell lipoma often requires the confirmation of MDM2 and CDK4 amplification [39]. Similarly, Rb1 expression analysis is valuable in differentiating well-differentiated from dedifferentiated liposarcoma, the latter often exhibiting Rb1 loss and aggressive clinical behavior [18,40,41]. Dedifferentiated liposarcoma demonstrates high metastatic potential, which is influenced by factors such as anatomical site, necrosis, and proliferative activity [6,42].

Therapeutic decisions are guided by histologic subtype, tumor burden and metastatic status [43,44,45]. In our cohort, most patients underwent surgical resection followed by chemotherapy with amputation reserved for advanced or recurrent extremity tumors. Despite these interventions, treatment options remain limited, and accurate diagnosis and risk stratification are critical due to the disease’s variable clinical course [18,31,46,47,48]. Tumor location has been proposed as an independent prognostic factor, particularly in retroperitoneal tumors where complete resection is challenging [49,50].

Systemic treatments such as doxorubicin, ifosfamide, gemcitabine and docetaxel are the medication commonly used for dedifferentiated liposarcoma [36]. However, the limited efficacy of drugs like trabectedin, eribulin, and pazopanib has driven several clinical trials exploring targeted therapies including CDK4 and MDM2 inhibitors alongside immunotherapeutic approaches [36]. Recognized for its radiosensitivity, myxoid liposarcoma responds well to multimodal therapy, and it is defined by FUS-DDIT3 gene fusion [51,52,53]. This genetic peculiarity of this intermediate-grade liposarcoma may hold promise for the use of FUS-DDIT3 as a diagnostic marker and therapeutic target [51,52,53]. Emerging research links this fusion with JAK-STAT signaling, suggesting potential responsiveness to JAK1/2 inhibitors [53,54].

In contrast, pleomorphic liposarcoma remains poorly understood due to its rarity, morphologic heterogeneity and lack of consistent biomarkers [55,56,57]. Standard treatment typically involves surgery and chemotherapy, with the role of preoperative radiotherapy still under investigation, especially in retroperitoneal presentations [58,59]. While several mutations—such as TP53, TOP2A, and CDH1—have been implicated in liposarcoma pathogenesis, no mutation has been conclusively linked to pleomorphic subtypes [60,61]. Current studies are exploring the role of gene rearrangements and mutations including NTRK, ROS1, PDL-1, and ALK with trials evaluating the efficacy of targeted therapies such as larotrectinib, tyrosine kinase inhibitors, and immune checkpoint inhibitors [62,63,64].

## 5. Conclusions

Histologic grade remains a key prognostic factor in liposarcoma with high-grade tumors—particularly grade 3—demonstrating significantly poorer overall survival. Proliferative activity also holds substantial prognostic value and can be assessed through both conventional microscopy and immunohistochemical evaluation using the Ki67 index. The Ki67 analysis not only correlates strongly with tumor grade but also provides valuable prognostic insights particularly in distinguishing between low- and high-grade malignancies. The integration of this immunohistochemical marker into diagnostic workflows may enhance the precision of risk stratification and may allow for more individualized patient management strategies. Furthermore, the progressive increase in proliferative activity from low- to high-grade tumors highlights the added value of Ki67 immunohistochemistry in refining prognostic assessment and tailoring follow-up intensity. In this context, the LEMON score—an integrative model combing mitotic index, histologic subtype and tumor necrosis—demonstrates promising clinical utility in stratifying patients by risk. This scoring system effectively differentiates between indolent and aggressive liposarcomas across tumor grades, reinforcing its potential role in guiding therapeutic strategies. Moreover, the heterogeneity in clinical outcomes, even among patients with similar histological liposarcoma variants, supports the growing recognition of immunohistochemical and molecular profiling in improving diagnostic accuracy and therapeutic planning. For instance, the evaluation of CDK 4, MDM2 and RB-1 expression along with Ki67 has shown potential in distinguishing tumors from different prognostic categories, guiding personalized care in liposarcoma management.

In summary, the findings of the present study, in correlation with the data available in the scientific literature, advocate for a multidimensional diagnostic approach for liposarcoma. Adequate evaluation systems should integrate histopathological, immunohistochemical and molecular parameters to refine prognostic assessment, inform therapeutic decisions and ultimately improve patients’ outcomes.

## Figures and Tables

**Table 1:** Overall survival in patients with liposarcoma based on the LEMON score.

	Statistic for High Risk (6–8)	Statistics for Low Risk (3–5)
Number of patients	51	48
1-year survival	56.86%	62.50%
3-year survival	27.45%	39.58%
5-year survival	11.76%	22.92%
Mean survival (months)	26.49	37.93
Median survival (months)	17.48	15.26

## Data Availability

The original contributions presented in this study are included in the article. Further inquiries can be directed to the corresponding author.
